# 3D Printing Manufacturing of Polydimethyl-Siloxane/Zinc Oxide Micro-Optofluidic Device for Two-Phase Flows Control

**DOI:** 10.3390/polym14102113

**Published:** 2022-05-22

**Authors:** Giovanna Stella, Matteo Barcellona, Lorena Saitta, Claudio Tosto, Gianluca Cicala, Antonino Gulino, Maide Bucolo, Maria Elena Fragalà

**Affiliations:** 1 Dipartimento di Ingegneria Elettrica, Elettronica ed Informatica dell’Università degli Studi di Catania, Viale Andrea Doria, 6, 95125 Catania, Italy; giovanna.stella@phd.unict.it (G.S.); maide.bucolo@unict.it (M.B.); 2 Dipartimento di Scienze Chimiche, Università degli Studi di Catania and INSTM Udr Catania, Viale Andrea Doria, 6, 95125 Catania, Italy; matteo.barcellona@phd.unict.it (M.B.); agulino@unict.it (A.G.); 3 Dipartimento di Ingegneria Civile ed Architettura, Università di Catania ed INSTM Udr Catania, Viale Andrea Doria, 6, 95125 Catania, Italy; lorena.saitta@phd.unict.it (L.S.); claudio.tosto@unict.it (C.T.); gcicala@unict.it (G.C.)

**Keywords:** polydimethyl-siloxane (PDMS), ZnO, microfluidic, 3D printing, surface functionalization

## Abstract

Tailored ZnO surface functionalization was performed inside a polydimethyl-siloxane (PDMS) microchannel of a micro-optofluidic device (*mofd*) to modulate its surface hydrophobicity to develop a method for fine tuning the fluid dynamics inside a microchannel. The wetting behavior of the surface is of particular importance if two different phases are used for system operations. Therefore, the fluid dynamic behavior of two immiscible fluids, (i) air–water and (ii) air–glycerol/water in PDMS *mofds* and ZnO-PDMS *mofds* was investigated by using different experimental conditions. The results showed that air–glycerol/water fluid was always faster than air–water flow, despite the microchannel treatment: however, in the presence of ZnO microstructures, the velocity of the air–glycerol/water fluid decreased compared with that observed for the air–water fluid. This behavior was associated with the strong ability of glycerol to create an H-bond network with the exposed surface of the zinc oxide microparticles. The results presented in this paper allow an understanding of the role of ZnO functionalization, which allows control of the microfluidic two-phase flow using different liquids that undergo different chemical interactions with the surface chemical terminations of the microchannel. This chemical approach is proposed as a control strategy that is easily adaptable for any embedded micro-device.

## 1. Introduction

Currently, the hydrodynamic of two-phase flows in a microchannel plays an important role in micro–nano technology, enabling the design of point-of-care devices in the biomedical field and micro-electrical–mechanical systems in chemical processes [1,2,3].

An open issue in this context is the design of control systems easily adaptable to different operative conditions and able to guarantee process reproducibility and reliability [4,5]. The studies presented in the literature are strictly related to specific experimental conditions, far from being a well-established framework that can drive flow control. Recently, some case studies have been presented in the literature using a system-on-a-chip (SoC) approach that embeds model predictive control strategies [6]. The SoC offers a high level of control and modularity, but its functionalities are strongly dependent on both integrated control logic and knowledge of the process model [7].

In this work, a chemical approach based on the treatment of the microchannel surface is presented as a control strategy that is easily adaptable for embedded micro-devices. The interaction between fluids and the microchannel surface was studied to investigate the possibility of slowing-down or accelerating the two-phase flows, generated by the interlaced sequence of two immiscible fluids at a microfluidics T-junction: at the T-junction, two immiscible liquids produce droplets whose movement is associated with periodic variation of the refractive index (RI) [8]. This approach was tested using a micro-optofluidic device (*mofd*), similar to those designed by the authors in a previous work [9], in which the microchannel and the micro-optical components for real-time flow detection are integrated.

Two-phase flows in the microchannel are determined by the wetting properties of the channel surface and, from this perspective, functionalization strategies are fundamental to tailor fluid dynamics at the micrometric scale. Among the materials used to fabricate microchannels, polydimethyl-siloxane (PDMS) is a long-lasting material largely used in microfluidic device fabrication due to fast prototyping by soft-lithography [10] and recently also 3D printing [11], due to its material properties, namely, biocompatibility, transparency and easy Complementary Metal-Oxide Semiconductors (CMOS) integration. Accordingly, compared with other microfluidic devices made of rigid materials, such as glass, silicon or ceramics, PDMS-based reactors guarantee easier manufacturing processes at low cost.

In recent decades, micro-optical components were also created using polydimethyl-siloxane (PDMS), and integrated with the microfluidics device, opening the opportunity of moving from the equipment used to perform the standard optical sensing procedures to their miniaturization into a single low-cost portable device [12,13,14].

Optical approaches to detect and control two-phase flows in microchannels offer the advantages of a wide range of measurement options being minimally invasive. In recent studies carried out by the authors, optical signals were used to classify and identify the two-phase flow inside the microchannel [15], to characterize the flow non-linearity [16], and for a real-time velocity detection [17].

The idea proposed in this work is to investigate the possibility of integrating a passive flow control within a micro-optofluidic device by PDMS surface treatment, thereby avoiding external devices, as used in the active control, and overcoming the need for specific microchannel geometry, as required, in the passive control.

Examples of active control are based on mechanical pumping, pneumatic pressure or electro-magnetic field [18]. A passive control, mainly used in capillary microchannels [19,20] can be implemented to generate a specific flow pattern, thus exploiting the geometrical properties of the microchannel and the physical properties of the fluids involved in the process, such as the hydrophobicity or the surface tension between the fluids and the walls of the channels. This approach does not require additional energy sources and does not increase the complexity and cost of the external equipment.

One of the main concerns relating to PDMS properties affecting microfluidic device performance is its hydrophobicity and low chemical resistance to many nonpolar organic solvents, and, to address these drawbacks, surface modification strategies are often adopted using a wide selection of functionalization approaches [21]. In particular, surface coating with inorganic structures might contribute to improving PDMS robustness although surface behavior modification must be expected [22,23,24,25]. The combination of inorganic nanomaterials with PDMS in microfluidic devices involves nanostructured ZnO, a semiconductor material which is non-toxic and biodegradable, and considered a versatile nanoplatform in many fields, including biosensing and bioimaging [26,27] and photocatalytic applications for water treatments [28].

Other authors deposited ZnO nanostructures on a PDMS microchannel with the aim of exploiting micro- or nanoscale surface roughness to tailor flow resistance inside the channel and control water mobility inside closed channels. However, most of the presented approaches use PDMS microfluidic devices with a silicon, quartz or glass base, thus requiring a photolithographic procedure to open the channel [29,30].

In this paper, a selective deposition of ZnO nanostructured coating was performed inside the PDMS channel, without altering the overall optical transparency, to modify the morphology and chemical composition of the surface. The proposed methodology differs from similar approaches reported in the literature where ZnO growth by CBD was achieved on silicon or quartz substrates preliminarily deposited with thin ZnO or other material deposited as a seed layer [31,32]. In this paper, we grow ZnO nanostructures directly on PDMS microchannels with an exclusive solution approach. This strategy has the advantage of being economical and does not require any additional lithographic step, thus resulting as suitable for disposable use to overcome any issues related to ZnO durability.

Chemical bath deposition (CBD) was integrated in the process flow used to fabricate the PDMS-based micro-optofluidic devices (*mofd*): in particular, ZnO nanorods were grown by CBD on a PDMS *mofd* by using a 3D printed mask to selectively limit the growth inside the microchannel.

To test the capability of these PDMS-ZnO-based devices, optical signals acquired in a specific test section of the microchannel were analyzed and compared with unmodified PDMS devices. The fluid dynamic study allowed us to unveil the key roles of surface roughness and chemistry on water/air and glycerol/air mobility.

## 2. Materials and Methods

### 2.1. Materials

Zinc acetate dihydrated (Zn(CH_3_COO)_2_ 2H_2_O) and ethylene diamine (NH_2_CH_2_CH_2_NH_2_) were purchased from Sigma Aldrich (St. Louis, MO, USA). The PDMS was the Sylgard 184 elastomer kit from Dow Corning. The commercial UV DLP Hard White resin was purchased from Photocentric Ltd. (Peterborough, UK) and is a liquid, high temperature resistant, photo-polymeric resin based on a proprietary mixture of acrylate monomers.

### 2.2. Zinc Oxide Chemical Bath Deposition on Polydimethyl-Siloxane

The PDMS devices were dipped for 1 h in an aqueous solution of zinc acetate dehydrate (Zn(CH_3_COO)_2_·2H_2_O, 99.999%, 0.1 M) and then heated overnight at 110 °C. The ZnO seed coated PDMS substrates were immersed in a nutrient bath containing an aqueous solution of zinc acetate dehydrate (0.05 M) and ethylenediamine (EDA, 0.05 M). After stirring at 90 °C for 3 h, the sample was rinsed with deionized water (DI) and dried at room temperature [33,34].

**Figure 1:**
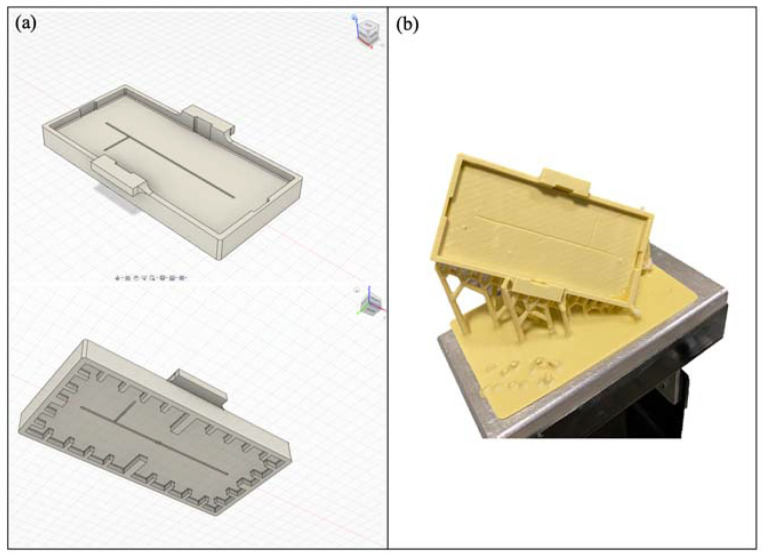
Customized mask for the PDMS device design using Autodesk^®^ Fusion 360 (**a**); 3D printed mask (**b**).

**Figure 2:**
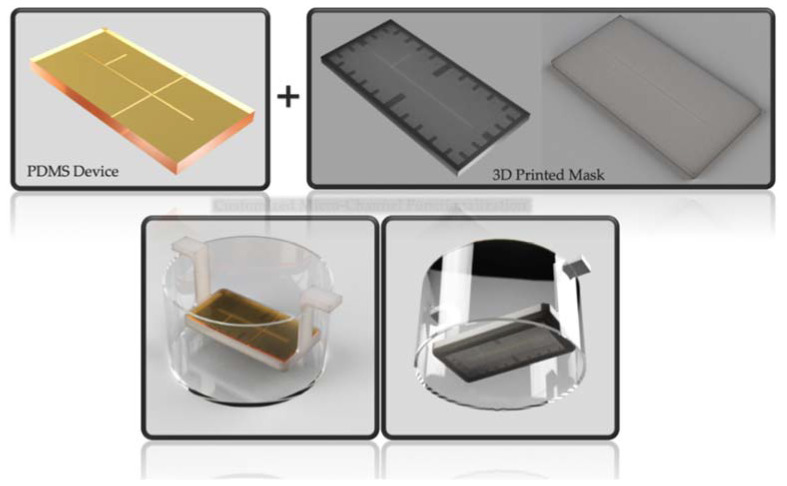
Rendering of the mask–device assembly suspended by a 3D printed support in the bath to grow ZnO inside the device’s channel.

To promote the ZnO growth exclusively in the PDMS device’s microchannel, first, a customized mask was designed using the 3D modeling software Autodesk^®^ Fusion 360 (Autodesk Inc., San Rafael, CA, USA) (Figure 1a). Next, it was 3D printed using an LCD 3D printer (LC Ceramic Precision, Photocentric Ltd., Peterborough, UK) with the Hard White resin as the material. The obtained final part is shown in Figure 1b. The mask–device assembly required immersion, keeping its surface at a fixed height where ZnO nanocrystals could grow. The 3D printed support shown in Figure 2 was designed to place the device at a predetermined height during the ZnO growth.

### 2.3. Contact Angle Measurements

Pristine and chemically modified PDMS surfaces were characterized by static water contact angle (θ) measurements, at room temperature, in air, using a Lite Optical Tensiometer TL100 (KSV, Helsinki, Finland) with an accuracy of ±3°. Briefly, 5 µL of Milli-Q water (resistivity 18.2 MΩ at 25 °C) drops were applied on the PDMS device surface with a calibrated micro-syringe, and measurements of θ were made on both sides of the two-dimensional projection of the droplet. Five different sets of measurements were performed on different surface portions of every sample to obtain statistically reliable results.

### 2.4. X-rays Photoelectron Spectroscopy

X-ray photoelectron spectra (XPS) were measured for pristine and chemically modified PDMS devices at a 45° take-off angle, relative to the surface sample holder, with a PHI 5600 Multi Technique System (Physical Electronics GmbH, Feldkirchen, Germany, base pressure of the main chamber 1 × 10^−8^ Pa) [35,36]. Samples placed on a molybdenum specimen holder were excited with the Al-Kα X-ray radiation using a pass energy of 5.85 eV. The instrumental energy resolution was ≤ 0.5 eV. Structures due to the Al-Kα X-ray satellites were subtracted from the spectra prior to data processing. XPS peak intensities were obtained after a Shirley background removal. Spectra calibration was achieved by fixing the Ag3d5/2 peak of a clean sample at 368.3 eV; this method turned the C1s main peak at 285.0 eV. Atomic concentration analysis was performed by considering the relevant atomic sensitivity factors.

### 2.5. Atomic Force Microscopy Measurements

The morphology of the pristine and chemically modified PDMS devices was observed by atomic force microscopy (AFM) using an NT-MDT Integra System instrument (Moscow, Russia). The noise level before and after each measurement was 0.01 nm. AFM characterizations were performed in a high-amplitude mode (tapping mode, resonance frequency 150 Hz) to avoid any possible modification of the grafted layer on the surfaces, caused by the interactions with the tip, whose nominal curvature radius was 10 nm. Surface roughness (RMS, Ra) was measured from 20 × 20 and 5 × 5 µm^2^ scans and was the average of at least three images scanned at different locations on the sample surface.

### 2.6. Scanning Electron Microscopy Measurements

Images were acquired using a field emission scanning electron microscope (FESEM, VP-Supra 550 FE-SEM (Zeiss, Oberkochen, Germany)) at accelerating voltages of 15 kV. Samples were sputtered with a thin gold layer to reduce the surface charging up.

### 2.7. The Micro-Optofluidic Device Design and Creation

**Figure 3:**
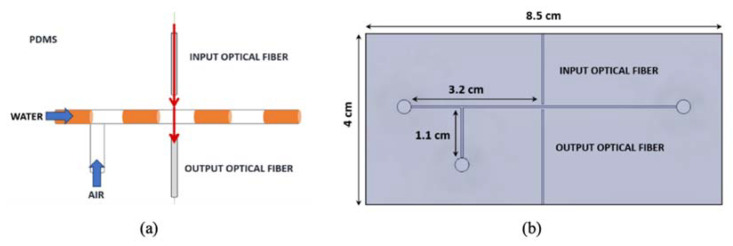
Working principle of the micro-optofluidic systems based on absorption (**a**); the CAD representation and measurements in a frontal perspective: the microfluidics T-junction and the optical fiber insertions (**b**).

The PDMS micro-optofluidic system used in this study exploits the phenomenon of light absorption for two-phase flow detection of immiscible fluids. Assuming that two fluids with quite different refraction indices flow in a microchannel and an incident laser beam interferes with them in a specific test section of the microchannel, it is possible to obtain a different light transmission based on which fluid is interfering with the laser beam at that moment. Thanks to the optical fiber insertion, the laser light is conveyed to a specific section of the investigated microchannel. The light travels through the sample and is detected by a second optical fiber aligned at the opposite side of the microchannel. The optical signal acquired is then correlated to the flow inside the microchannel. A schematic of the working principle is shown in Figure 3a, while the CAD representation of the PDMS micro-optofluidic system is presented in Figure 3b.

The *mofd* was created using a master–slave approach based on the use of inkjet 3D printing techniques. The CAD was printed using a photo-sensitive resin, and the surfaces treated to avoid any type of reticulation of the resin in contact with the PDMS [11]. The master mold was printed using a professional inkjet printer, model Objet260 Connex1, Stratasys (Rheinmnster, Germany). The material used for the mold was VeroWhitePlus, and FullCure705 (OVERMACH S.p.A., Parma, Italy) was used as support. The silicone and the curing agent of PDMS were mixed together according to the (10:1) proportion for the device layer and to the (5:1) proportion for the bulk cover layer. After degassing, the PDMS was poured into the master and placed in an oven at 50 °C for 24 h. Finally, the PDMS devices were peeled from the master and the device bound with a 0:5 mm thick bulk by a reversible binding procedure.

### 2.8. Experimental Apparatus

**Figure 4:**
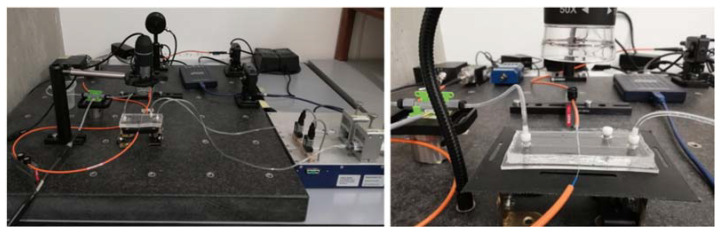
Pictures of the experimental setup used for the two-phase flow detection.

An interlacing sequence of air and water was generated by pumping liquid and air at the inlet of the T-junction of the PDMS micro-optofluidic device. The experimental set-up for the two-phase flow monitoring in the designed investigation area of the micro-optofluidic device is shown in Figure 4.

Two syringe pumps (neMESYS by Cetoni Gmbh, Münster, Germany) were connected to the two channel inlets and different flow rates were imposed. The input light source was a laser system (Rgb NovaPro Laser 660-125, Lasersystems, Kelheim, Germany) that generates a light beam with a wavelength of 600 nm. The light intensity variation was acquired by means of a photodiode (PDA 100A, Thorlabs, Newton, NJ, USA, gain used 40 dB) and the signal received was acquired by a PC oscilloscope (Picoscope 2204A, Pico Technology, Cambridgeshire, UK), with a sampling frequency of 1.5 KHz. A digital USB microscope was placed above the device to simultaneously conduct image recording of the process.

In the experimental campaign, a total of 24 experiments, 12 per mof-device {*PDMS-mofd* and the *ZnO-PDMS-mofd*}, were carried out to evaluate the variation between the two-phase flow velocities inside the microchannel.

The experiments were performed using a different device for each experimental condition, thus demonstrating the reproducibility of the functionalization strategy.

To evidence how the chemical treatment affects the flow dynamics, the fluids considered were air, water and a mixture of glycerol–water (50% *w/w*): investigated two-phase flows were (i) air–water (FLOW1) and (ii) air–glycerol–water (FLOW2).

The data acquired were pre-processed by a low-pass filter with a 40 Hz cut-off frequency and a signal smoothing procedure. In the processing phase, the 24 signals acquired were analyzed both in time and frequency domains to automatically detect the frequency of the air–liquid passages. The developed methodology was widely used in previous works [16].

By optical fiber insertion, it was possible to capture the variation in the luminosity during the two-phase passage, due to the difference between the refraction index (*n*) of the chip material PDMS (*n*_PDMS_= 1.41), air (*n*_air_ = 1), water (*n*_water_ = 1.3) and mixture (*n*_(Glycerol–Water)_ = 1.39). Thanks to this phenomenon, the air and liquid passages were detected in the optical signal on two brightness levels. The top level revealed the liquid presence, the low level revealed the air passage, and the two peaks revealed the air in front and at rear.

## 3. Results and Discussion

### 3.1. Surface Characterization of Polydimethyl-Siloxane and Polydimethyl-Siloxane/Zinc Oxide Slubs

**Figure 5:**
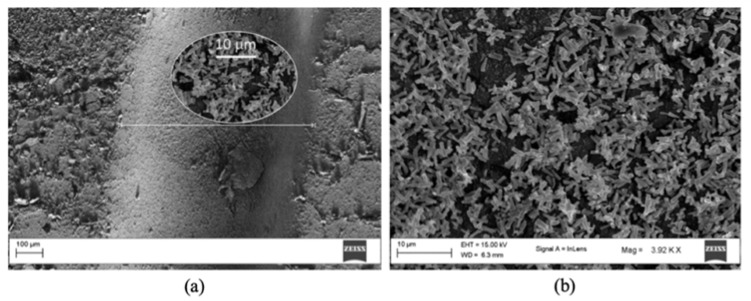
SEM image of the channel region of the PDMS *mofd* after ZnO growth (**a**); high resolution image of ZnO nanostructures inside channel (**b**).

Fluid flow in the microchannel and fluid interaction with microchannel surfaces are critical for *mofd* performance. Wettability of the microchannel surface governs the dynamic of fluids through a balance of cohesive forces within the liquid and adhesive forces between the liquid and its surroundings. Accordingly, a control of hydrophobicity allows for an improved versatility of these systems. In particular, the possibility of modulating the surface hydrophobicity by growing ZnO nanostructured layers inside the device’s channels opens the way to fluid dynamic control at a local scale and thus, to the development of novel microfluidic devices [37]. Therefore, coating the main microchannel of the PDMS device with a nanostructured layer of ZnO rods is expected to influence the dynamics of a two-phase fluid, due to both liquid–solid chemical affinity and morphological factors relating to the roughness control. In this paper, two PDMS-based *mofd* were compared: a bare reference (PDMS) with uncoated surfaces and a ZnO treated sample (ZnO-PDMS) with microchannel coated surfaces. Chemical bath deposition performed at 90 °C in aqueous nutrient solution was considered as a suitable technique to grow ZnO nanostructured layers composed of nanorods inside the PDMS channels, as shown in Figure 5.

The low-resolution SEM image (Figure 5a) clearly shows the channel region of the PDMS component, while Figure 5b provides detail of the ZnO nanorods’ dense coverage of the entire channel area.

**Figure 6:**
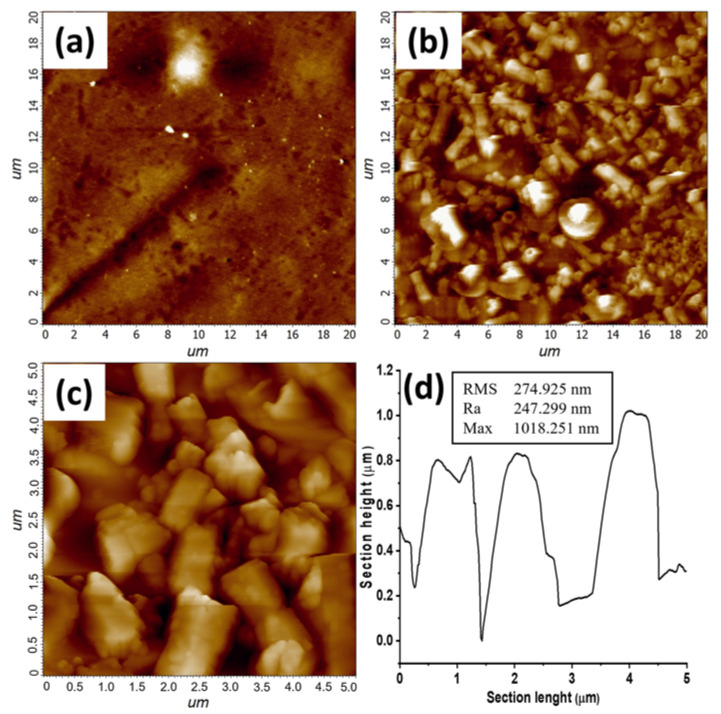
AFM images of the PDMS-ZnO surface: scale bar 20 × 20 µm^2^ (**a**), and 5 × 5 µm^2^ (**b**); 20 × 20 µm^2^ AFM image of bare PDMS surface (**c**); section analysis and topographical information (**d**).

ZnO nanorods were well visible in the AFM measurements (Figure 6) which revealed the hexagonal structure typical of the wurtzitic phase. It was noteworthy that a bimodal growth of ZnO nanostructures was detected on the PDMS surface (Figure 6a,b): large hexagonal and misoriented micro-rods on top of a layer of smaller c-axis oriented nanorods. Surface morphology of bare PDMS is shown for comparison in Figure 6c.

Related topographical information, expressed in terms of roughness (root mean square (RMS) and mean heights (Ra)) and maximal heights of the structures [38], are reported in Figure 6d. Mean lateral particle dimensions, estimated by ImageJ software, were 1.6 μm, with a minimum value of 670 nm and a maximum value of 2.5 μm.

The presence of ZnO inside the microchannel on the PDMS surface was also confirmed by XPS analysis. Table 1 shows the XPS atomic concentration analysis for two representative PDMS and ZnO-PDMS devices.

**Figure 7:**
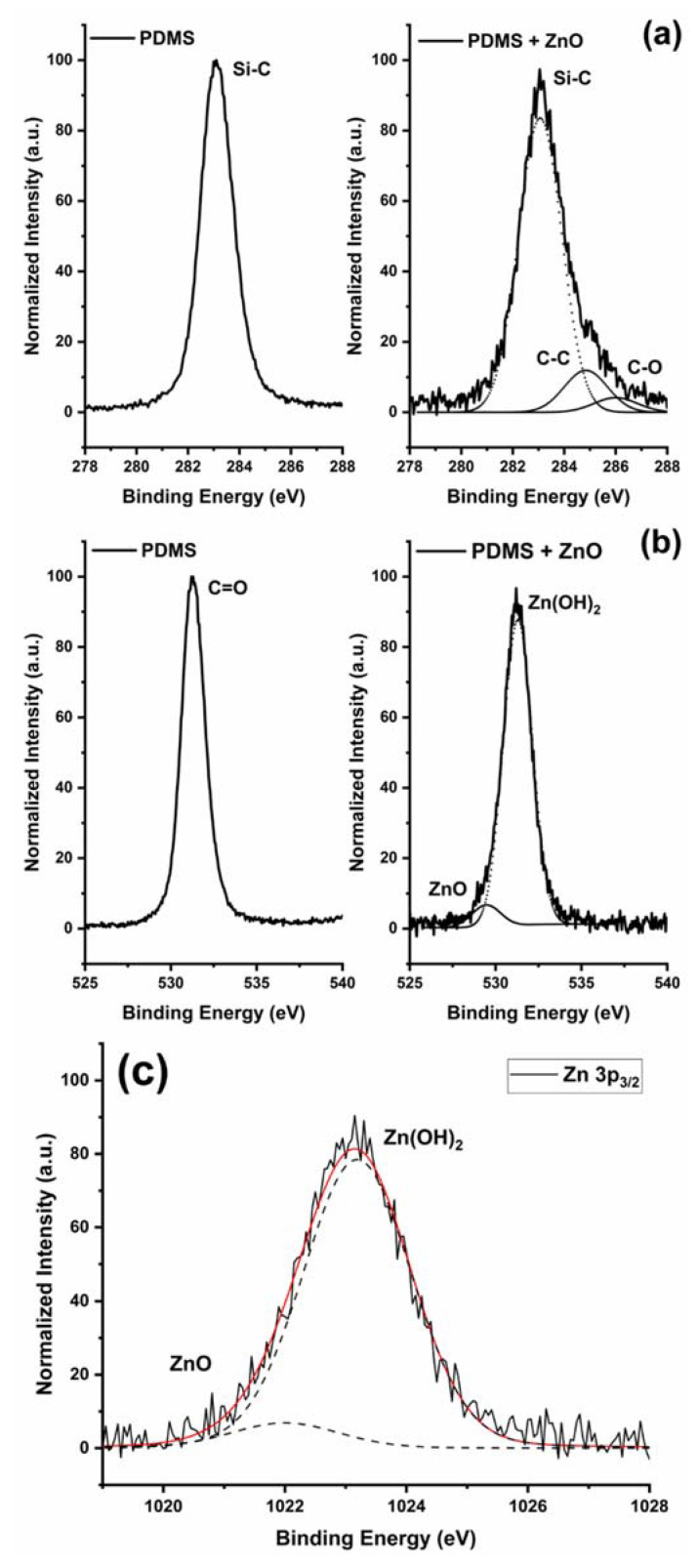
PDMS reference (dotted lines) and PDMS-ZnO treated slub (solid lines) spectral comparison of C1s (**a**), O1s (**b**), and Zn2p3/2 (**c**), X-ray photoelectron peaks.

By comparing the C1s XP spectra before and after the CBD growth (Figure 7a), a band broadening was well evident and additional components to the main Si-C (at 283.7 eV) were evident at high binding energy and were associated with C-O bonds (286.2 eV) and C = O bonds (288 eV). The O1s peak after the ZnO growth was characterized by a tail at 529.6 eV associated with O_2_^−^ and OH^−^ ions in the defective sublattice of ZnO (Figure 7b). It was noteworthy to observe that the shape of the Zn2p3/2 peak revealed two components (Figure 7c), one associated with ZnO at 1022 eV [39] and a second at 1023.2 eV associated with a zinc hydroxide phase, ε-Zn(OH)_2_, formed during the CBD growth in the alkaline solution [40,41].

### 3.2. Two-Phase Microfluidic Flow Characterization

Flow’s fluid dynamic evaluation was carried out by varying both the hydrodynamic pressure at the inlet of the chip and the power of the laser light used to visualize the process, as summarized in Table 2.

The hydrodynamic pressure was set using three input flow rate values (f), keeping the same velocity for air (V_air_) and liquid (V_lq_). The power of the laser was set using two levels of intensity (P). After turning on the laser, the same power intensity was maintained for 60 s for each hydrodynamic condition (f).

**Figure 8:**
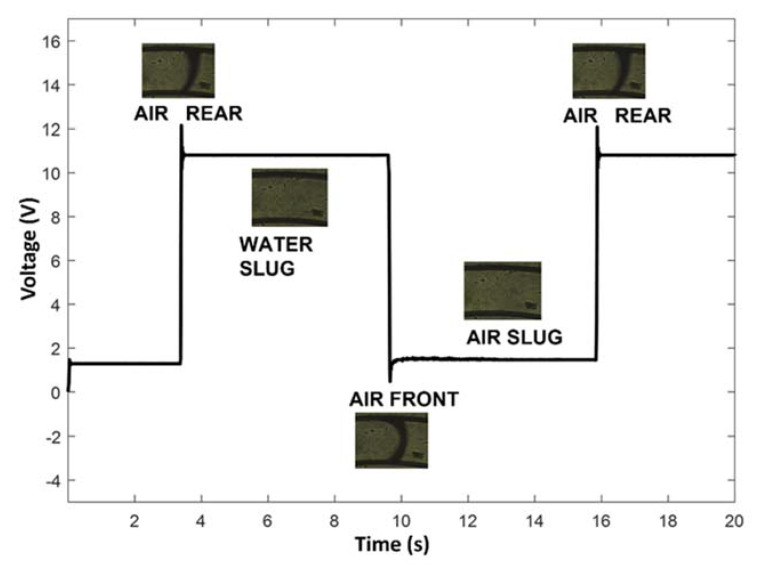
Optical signal acquired in the PDMS *mofd* correlated to the FLOW 1 passage in the EXP2 conditions (flow rate 0.1 mL/min).

Figure 8 shows the optical signal and related CCD video frames acquired during an air–water passage in the test section of the micro-optofluidic device.

**Figure 9:**
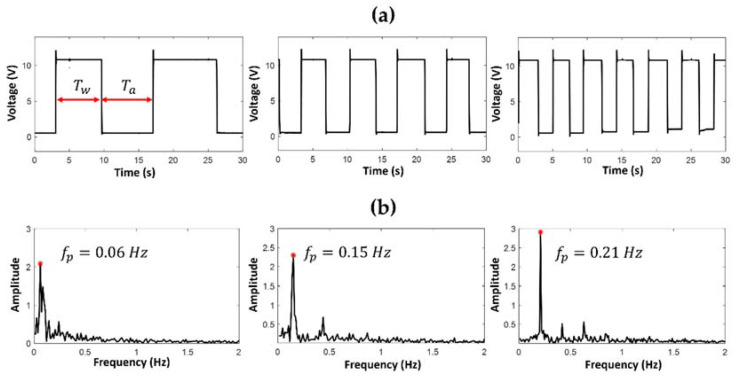
Optical acquisition for FLOW1 in PDMS *mofd* using EXP1 combinations, flow rate V_air_ = V_water_ = (0.1, 0.2, 0.3) mL/min: (**a**) the slug passage signals, and (**b**) the spectra.

**Figure 10:**
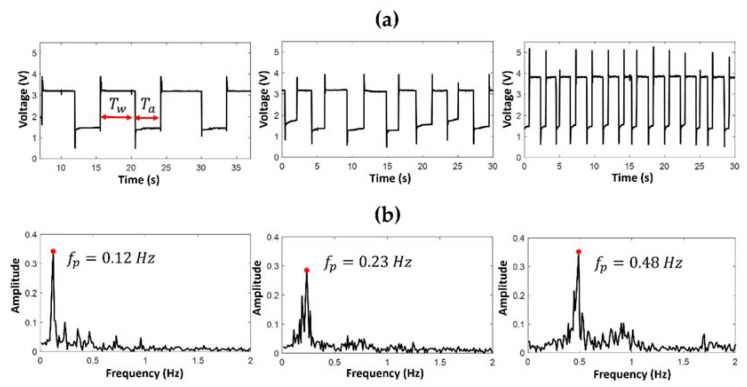
Optical acquisition for FLOW1 in ZnO-PDMS mofd using EXP1 combinations flow rate Vair = Vwater (0.1, 0.2, 0.3) mL/min: (**a**) the slug passage signals, and (**b**) the spectra.

Figure 9 and Figure 10 show the trends and spectra of the optical signals acquired using a photodetector, respectively, in the PDMS *mofd* and ZnO-PDMS *mofd* using FLOW1 (air–water) in the EXP1 combinations.

The parameters *T_w_* and *T_a_* indicated in Figure 9a and Figure 10a identify the duration of the liquid and air passage, respectively: the value of the frequency peak (*fp)* reported in Figure 9b and Figure 10b is related to the inter-distance of air–water (or glycerol/water) passage.

This frequency peak was used to calculate the average period (T_period_) of the flow passage:

T_period_ = 1/f_p_ = <T_w_> + <T_a_> (1)

By visual inspection, it was evident how the performances of the two devices were different: the number of FLOW1 passages in 30 s in the PDMS *mofd* were fewer than in the ZnO-PDMS *mofd*. A similar behavior was observed when FLOW1 was tested in the EXP1 conditions.

**Figure 11:**
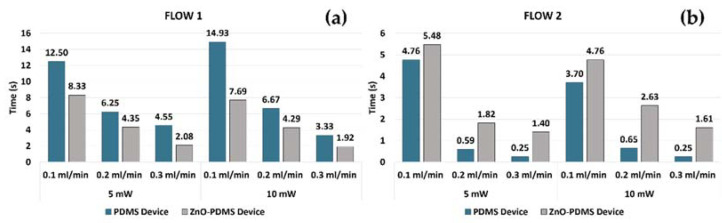
T_period_ measured in EXP1 and EXP2 conditions for FLOW1 (**a**), and FLOW2 (**b**), in PDMS *mofd* and ZnO-PDMS *mofd*.

The period of the air–liquid passage (T_period_) in the area investigated using both PDMS and ZnO-PDMS *mofd* devices are reported in Figure 11.

The two bar graphs reported in Figure 11a,b summarize the obtained results for the two fluid combinations (FLOW1 and FLOW 2) in EXP1 and EXP2 conditions. To note, the T_period_ of FLOW2 was always lower (faster flow) than FLOW1 (slower flow), disregarding the microchannel treatments and the experimental conditions, as expected when considering the glycerol–water solution viscosity and density.

It is noteworthy that, in Figure 11a, the T_period_ obtained for FLOW1 in the same experimental conditions (i.e., laser power and input flow-rate) was lower in the ZnO-PDMS *mofd* (faster flow) than in the PDMS-*mofd* (slower flow). This means that the ZnO surface functionalization led to an increase in the velocity of FLOW1. This finding accords with the contact angle (CA) measurements of the PDMS surface after ZnO coating: in particular, the contact angle increased from 108° to 133°, and such hydrophobicity enhancement was related to the increase of surface roughness due to the presence of ZnO microstructures [42], causing enhanced water flow inside the channel [24].

Conversely, Figure 11b reports the period of the air–liquid passage (T_period_) related to FLOW2, in the same experimental conditions: the T_period_ is greater in the ZnO-PDMS *mofd* (slower flow) than the PDMS *mofd* (faster flow). Glycerol–water solution has a polarity lower than water and, in addition, is a trihydric alcohol able to form an extended hydrogen-bonded network. Accordingly, the observed behavior of FLOW2 in a ZnO-PDMS device can be attributed to the interaction of glycerol with the ZnO rods present in the microchannel and related network of H-bond formation between the surface atomic moieties of the deposited oxide [43].

In order to quantify the difference in the performance of the two devices, the percentage of change for each experimental condition was computed follows:

(2) $$\Delta \%=\left(\left(\frac{Xf}{Xi}\right)*100\right)-100$$

where *Xf* is the value of T_period_ measured for the PDMS-*mofd* device and Xi is for the T_period_ in the ZnO-PDMS *mofd* device.

**Figure 12:**
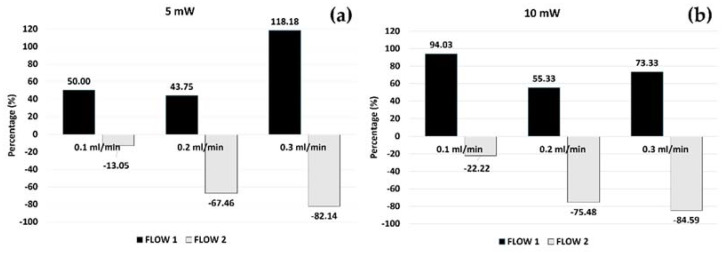
The percentage of change (Δ%) of T_period_ measured in the flow passage using the PDMS *mofd* and ZnO-PDMS *mofd* per experimental condition (EXP1 and EXP2) for (**a**) FLOW1 and (**b**) FLOW2.

Figure 12 reports the percentage (Δ%) values obtained.

On one hand, as expected, the Δ% obtained for FLOW1 was positive, underlining that the passage of FLOW1 was faster in the ZnO-PDMS *mofd* than in the PDMS *mofd.* On the other hand, the Δ% obtained for FLOW2 was negative due to the slower flow passage in the ZnO-PDMS *mofd* than the PDMS-*mofd.* Additionally, it was observed that this percentage variation was highly sensitive to the experimental conditions (input flow rate and the laser power). In particular, for FLOW2, the increase in the Δ% values following the increase in the input flow rates for both laser power conditions was much more regular than for FLOW1. That could be foreseen coherently with the higher level of instability of the air flow and the stabilization effect of the glycerol correlated to its density value (ρ = 1261 kg/m^3^) with respect to water (ρ = 1000 Kg/m^3^).

These results evidence the possibility of increasing the level of control of the flow inside the microchannel by combining the external forces (pressure strength and laser power) with a low-cost chemical treatment of the microchannel surface.

We continue to study the role of ZnO degradation on device performance as we are aware of drawbacks associated with ZnO solubility that can affect material functionality [40].

## 4. Conclusions

In this paper we studied the fluid dynamic of two PDMS micro-optofluidic devices (*mofds*), a bare PDMS reference, and a modified device with a ZnO nanorod coating of the microchannel, obtained by integration of CDD process with a master–slave PDMS manufacturing procedure. The two-phase flow detection was performed with an optical monitoring system allocated in the test section of the devices. After an optical acquisition processing phase, a post-processing phase was performed in time and frequency domain to characterize the fluid–laser interaction in the microchannel. Two different comparisons were performed for each device considering the two immiscible fluids. This analysis showed that the used two-phase fluids interacted differently with the microchannel of the investigated devices. In particular, the two-phase air–water flow was faster in the ZnO-PDMS mofd than in the PDMS mofd while the air–glycerol flow (FLOW2) was slower in the ZnO-PDMS mofd than in the PDMS mofd.

The results presented in this paper allow an understanding of the potential of ZnO functionalization, which enabled us to control the microfluidic two-phase flow in an indirect manner. The results show that even by changing the type of fluid in the device, we can speed up or slow down the process, thus evidencing the possibility of acquiring a passive control of the two-phase flow velocity by a chemical functionalization of the PDMS surface.

## Figures and Tables

**Table 1:** XPS atomic concentration analysis for two representative PDMS and ZnO-PDMS *mofd* devices.

Sample	C%	O%	Si%	Zn%	N%
PDMS	51.5	28.6	19.9	0	0
ZnO-PDMS	52.5	29.4	7.3	4.4	6.4

**Table 2:** Experimental campaigns carried out, varying the power of the laser used to light the process and the hydrodynamic pressure, for both the PDMS *mofd* and ZnO-PDMS *mofd* for both fluid combinations of air–water (FLOW1) and air–glycerol–water (FLOW2).

	Laser Light Power (mW)	Vair = Vwater Flow Rate (mL/min)
EXP1	5	0.1–0.2–0.3
EXP2	10	0.1–0.2–0.3
